# Basic host response parameters to classify mortality risk in COVID-19 and community-acquired pneumonia

**DOI:** 10.1038/s41598-024-62718-4

**Published:** 2024-06-03

**Authors:** Rosario Menéndez, Raúl Méndez, Paula González-Jiménez, Ana Latorre, Soledad Reyes, Rafael Zalacain, Luis A. Ruiz, Leyre Serrano, Pedro P. España, Ane Uranga, Catia Cillóniz, Andrea Gaetano-Gil, Borja M. Fernández-Félix, Luis Pérez-de-Llano, Rafael Golpe, Antoni Torres

**Affiliations:** 1 Pneumology Department, La Fe University and Polytechnic Hospital, Avda. Fernando Abril Martorell 106, 46026 Valencia, Spain; 2 Respiratory Infections, Health Research Institute La Fe (IISLAFE), Valencia, Spain; 3 University of Valencia, Valencia, Spain; 4 Centro de Investigación Biomédica en Red de Enfermedades Respiratorias (CIBERES), Instituto de Salud Carlos III, Madrid, Spain; 5 Pneumology Department, Cruces University Hospital, Barakaldo, Spain; 6 Department of Immunology, Microbiology and Parasitology, Facultad de Medicina y Enfermería, Universidad del País Vasco/Euskal Herriko Unibertsitatea UPV/EHU, Leioa, Spain; 7 Pneumology Department, Galdakao-Usansolo Hospital, Galdacano, Spain; 8 University of Barcelona, Barcelona, Spain; 9 Pneumology Department, Hospital Clinic of Barcelona, Barcelona, Spain; 10 August Pi i Sunyer Biomedical Research Institute (IDIBAPS), Barcelona, Spain; 11 Clinical Biostatistics Unit, Hospital Universitario Ramón y Cajal (IRYCIS), Madrid, Spain; 12 Centro de Investigación Biomédica en Red en Epidemiología y Salud Pública (CIBERESP), Instituto de Salud Carlos III, Madrid, Spain; 13 Pneumology Department, Lucus Augusti University Hospital, Lugo, Spain

**Keywords:** COVID-19, Pneumonia, Host response, Biomarkers, Mortality, Biomarkers, Translational research

## Abstract

Improved phenotyping in pneumonia is necessary to strengthen risk assessment. Via a feasible and multidimensional approach with basic parameters, we aimed to evaluate the effect of host response at admission on severity stratification in COVID-19 and community-acquired pneumonia (CAP). Three COVID-19 and one CAP multicenter cohorts including hospitalized patients were recruited. Three easily available variables reflecting different pathophysiologic mechanisms—immune, inflammation, and respiratory—were selected (absolute lymphocyte count [ALC], C-reactive protein [CRP] and, SpO_2_/FiO_2_). In-hospital mortality and intensive care unit (ICU) admission were analyzed as outcomes. A multivariable, penalized maximum likelihood logistic regression was performed with ALC (< 724 lymphocytes/mm^3^), CRP (> 60 mg/L), and, SpO_2_/FiO_2_ (< 450). A total of 1452, 1222 and 462 patients were included in the three COVID-19 and 1292 in the CAP cohort for the analysis. Mortality ranged between 4 and 32% (0 to 3 abnormal biomarkers) and 0–9% in SARS-CoV-2 pneumonia and CAP, respectively. In the first COVID-19 cohort, adjusted for age and sex, we observed an increased odds ratio for in-hospital mortality in COVID-19 with elevated biomarkers altered (OR 1.8, 3, and 6.3 with 1, 2, and 3 abnormal biomarkers, respectively). The model had an AUROC of 0.83. Comparable findings were found for ICU admission, with an AUROC of 0.76. These results were confirmed in the other COVID-19 cohorts Similar OR trends were reported in the CAP cohort; however, results were not statistically significant. Assessing the host response via accessible biomarkers is a simple and rapidly applicable approach for pneumonia.

## Introduction

The COVID-19 pandemic excessively burdened health systems worldwide, affecting millions of people. The scientific community, in response, shared an objective to develop models that would recognize disease subtypes or endotypes in patients with varying susceptibility to determine clinical evolution^1^. To date, several sophisticated omics-based studies have been done to provide undeniably informative knowledge about both the disease’s complex pathogenic mechanisms and the intricate host response^2–4^. Furthermore, multiple scores incorporating many clinical variables have been proposed; however, most require an on-line calculator^5^. The difficulty in stratifying patient subtypes depends on causal microorganism factors, such as SARS-CoV-2 or others; the heterogeneity of the host; and the eventual outcome of the interplay occurring between the aforementioned elements.

In pneumonia, improved phenotyping considering host response has been encouraged. Innate and adaptive responses could provide insights that contribute to individualized severity assessment and management^6^. In host response, there are three main basic components to determine, two of which are common in all infections—immune cells and inflammatory response—and the third is related to the lung—gas exchange function. From a very practical perspective, these components are measurable by quantifiable biomarkers/parameters that reflect a specific pathophysiologic area: absolute lymphocyte count (ALC), C-reactive protein (CRP), and peripheral blood oxygen saturation (SpO_2_)/fraction of inspired oxygen (FiO_2_). Indeed, lymphopenia was consistently reported in severe episodes during the pandemic. Similarly, in community-acquired pneumonia (CAP), lymphopenia at admission has been shown to double the risk of mortality^7,8^. CRP is one of the most frequent acute phase reactants available in the emergency department (ED) in hospitals and has also been studied in SARS-CoV-2 pneumonia. Oxygen saturation that indicates respiratory failure is considered one of the strongest predictors of deterioration^9^.

We hypothesized that a simple and feasible multidimensional approach that would require blood analyses and oxygen saturation, and include assessing three physiologic host response areas—inflammation, immune, and functional—could offer useful information during an initial evaluation of patients with either SARS-CoV-2 pneumonia or possibly CAP due to other causal microorganisms. In that case, patients could be classifiable per risk of mortality and clinical deterioration regardless causal microorganism.

The aim of the study was to estimate the effect of a basic and rapidly applicable tool based on ALC, CRP, and SpO_2_/FiO_2_ in addressing patient stratification per mortality risk or disease progression. A secondary aim was to evaluate this tool in CAP not caused by SARS-CoV-2.

## Methods

### Design and patients

A retrospective and multicenter study was designed. The COVID-19 cohort included patients admitted across four Spanish hospitals (La Fe University and Polytechnic Hospital in Valencia; Cruces University Hospital in Barakaldo; Galdakao-Usansolo Hospital in Galdacano; and Hospital Clinic of Barcelona) between March and May 2020. Inclusion criteria comprised clinical symptoms and a confirmed microbiologic diagnosis of SARS-CoV-2 by reverse transcription polymerase chain reaction (RT-PCR) testing on nasopharyngeal swabs. Exclusion criteria included those patients transferred from other medical facilities and under the age of 18 years. This study was conducted in accordance with the amended Declaration of Helsinki. The study was approved by the Biomedical Research Ethics Committee of La Fe University and Polytechnic Hospital (2020-122-1). Written informed consent was waived given the non-interventional nature of the study.

A second multicenter cohort with COVID-19 patients was included (hereafter referred to as the COVID-19 2020 validation cohort). This cohort comprised patients from across eight hospitals in Galicia who were diagnosed and admitted to hospital between March 1st and April 24th 2020. Inclusion and exclusion criteria were the same as in the first cohort. The Ethics Committee of Galicia (Cod. 2020/239) approved the study for the validation cohort.

A third cohort with COVID-19 patients admitted between 2022 and 2023 was included (hereafter referred to as the COVID-19 2022–23 validation cohort). This cohort comprised patients from La Fe University and Polytechnic Hospital who were diagnosed and admitted to hospital between January 1st 2022 and December 31st 2023 when omicron variant was circulating and population was highly vaccinated. Inclusion and exclusion criteria were the same as in the first cohort.

A fourth cohort (hereafter referred to as the CAP cohort) was considered in order to assess the generalization of the biomarkers’ effect on patients without COVID-19 yet presenting pneumonia. The cohort included those patients admitted to the same Spanish hospitals for CAP before December 2019. Patients recruited for this cohort met inclusion criteria if they received a diagnosis for pneumonia based on a new radiologic infiltrate and the presence of at least two compatible clinical symptoms. Exclusion criteria included admission within the previous 15 days, nursing home residency, and immunosuppressed patients. The study was approved by the ethics committee of each hospital, and patients signed an informed consent (2013/0204).

Demographics, comorbidities, treatments, and laboratory data were collected at baseline (emergency department admission), including those variables of interest for evaluating host response areas at admission: ALC (immune); CRP (inflammatory); and SpO_2_/FiO_2_ (respiratory). The main outcome was in-hospital mortality due to any cause. The secondary outcome analyzed was intensive care unit (ICU) admission.

### Statistical analysis

Patients were classified according to in-hospital mortality (alive or dead). Categorical variables under study were presented as absolute frequency and percentage, whilst quantitative variables as median and interquartile range (IQR). Differences between the two analysis groups were evaluated using the Wilcoxon rank-sum and Chi-square tests, as appropriate. Tables of association between biomarkers and in-hospital mortality were made, represented by absolute frequency, percentage and a 95% confidence interval (CI). There were 93.8% of cases with complete data, so multiple imputation was not performed.

A multivariable, penalized maximum likelihood logistic regression was performed^10^. Lymphocyte count, CRP, and SpO_2_/FiO_2_ were selected a priori as predictors of in-hospital mortality, as they had been identified as clinically relevant in the literature. A cut-off point was selected for each of the biomarkers based on former studies. The cut-off value for lymphocyte count was 724 cells/mm^3^. Patients with CAP presenting < 724 lymphocytes/mm^3^ at admission had been identified with having a higher risk of mortality^8,11^. Cut-off values for CRP and SpO_2_/FiO_2_ were 60 mg/L and 450, respectively. The latter is equivalent to an SpO_2_ < 94% in room air per the definition of severe COVID-19 by the Infectious Diseases Society of America (IDSA)^12^, and the cut-off point for CRP was selected from a prior study as we have found that < 60 mg/L was independently associated with low-risk for mortality^13^. Patients were grouped into four risk categories per the number of altered biomarkers in accordance with cut-off values (normal, or one, two or all abnormal markers). The estimate effect of each risk group was adjusted by sex and age, and represented as odds ratios (OR) and CI 95%. The reference was the normal marker group.

For the different biomarkers, survival curves of in-hospital mortality and ICU admission were estimated using the Kaplan–Meier method and compared by the log-rank test. All analyses were performed using STATA/IC 16.1 software (Stata Corporation, College Station, TX, USA).

### Ethics approval and consent to participate

This study was conducted in accordance with the amended Declaration of Helsinki. The study of the first cohort was approved by the Biomedical Research Ethics Committee of La Fe University and Polytechnic Hospital (2020-122-1). Written informed consent was waived given the non-interventional nature of the study. The Ethics Committee of Galicia (Cod. 2020/239) approved the study for the validation cohort. The CAP cohort study was approved by the ethics committee of each participant hospital, and patients signed an informed consent (Biomedical Research Ethics Committee of La Fe University and Polytechnic Hospital 2013/0204).

## Results

**Figure 1:**
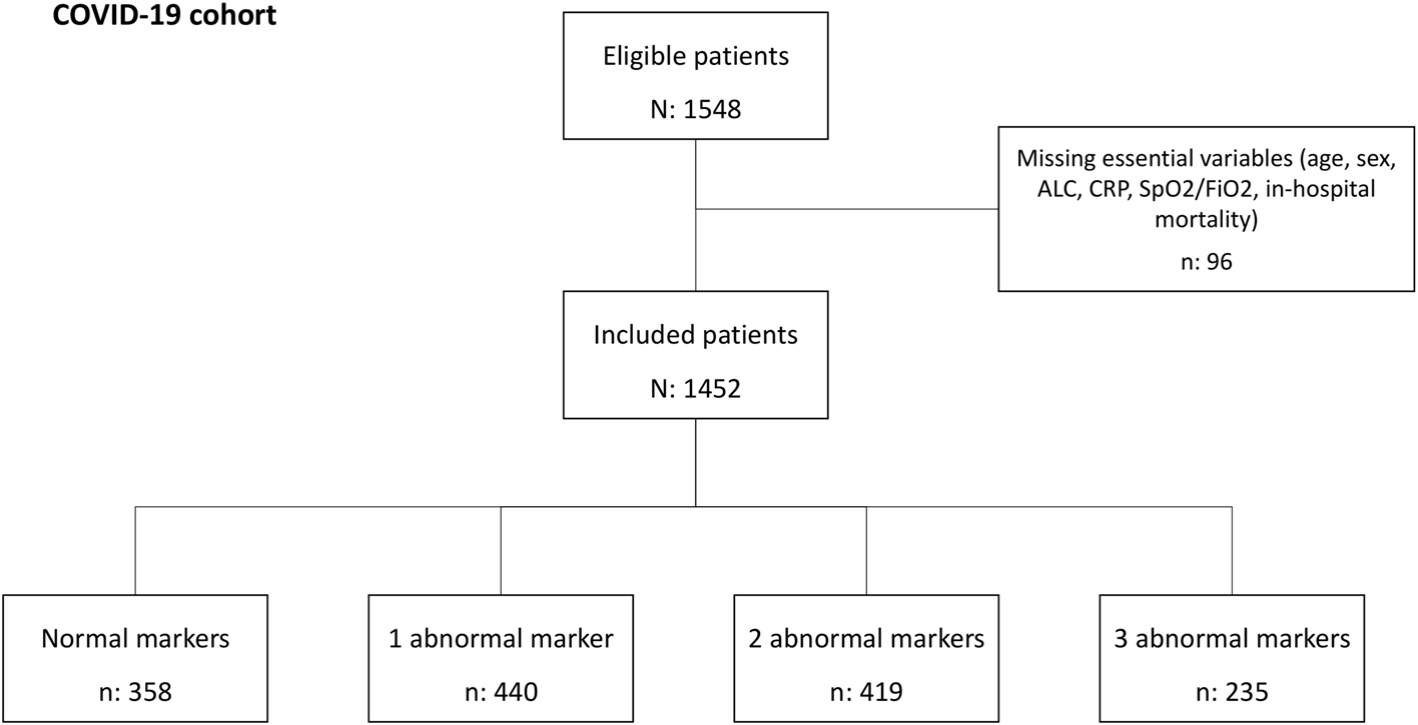
Study Flowchart. *ALC* absolute lymphocyte count; *CRP* C-reactive protein; *SpO*_*2*_*/FiO*_*2*_ peripheral blood oxygen saturation/fraction of inspired oxygen.

A total of 1548 patients were included in the COVID-19 cohort. Of these, 1452 (93.8%) had complete data for the selected variables (age, sex, ALC, CRP, and SpO_2_/FiO_2_) and main outcome under study (in-hospital mortality) (Fig. 1). Table 1 details baseline characteristics according to in-hospital mortality. Briefly, the patients who died were older, predominantly male, and with more comorbidities. In addition, they had lower levels of ALC and SpO_2_/FiO_2_, and higher levels of CRP. Supplementary Table 1, baseline characteristics are shown according to the number of markers (ALC, CRP, and SpO_2_/FiO_2_) altered.

**Table 1:** Baseline characteristics of COVID-19 cohort according to in-hospital mortality.

Variables	Alive (n = 1237)	Deceased (n = 215)	*P*-value
Demographics			
Age	63 (51–74)	80 (71–86)	< 0.001
Sex			
Female	501 (40.5%)	70 (32.6%)	0.028
Male	736 (59.5%)	145 (67.4%)	
Smoking	Missing N = 109	Missing N = 38	
No smokers	760 (67.4%)	125 (70.6%)	
Current smokers	74 (6.6%)	7 (4.0%)	0.380
Ex-smokers	294 (26.1%)	45 (25.4%)	
Comorbidities			
Arterial hypertension			
No	670 (54.2%)	52 (24.2%)	< 0.001
Yes	567 (45.8%)	163 (75.8%)	
Diabetes	Missing N = 3	Missing N = 0	
No	1005 (81.4%)	139 (64.7%)	< 0.001
Yes	229 (18.6%)	76 (35.3%)	
Dyslipidemia			
No	802 (64.8%)	107 (49.8%)	< 0.001
Yes	435 (35.2%)	108 (50.2%)	
Chronic heart disease			
No	1055 (85.3%)	131 (60.9%)	< 0.001
Yes	182 (14.7%)	84 (39.1%)	
Chronic renal disease	Missing N = 302	Missing N = 47	
No	851 (91.0%)	119 (70.8%)	< 0.001
Yes	84 (9.0%)	49 (29.2%)	
Chronic liver disease			
No	1203 (97.3%)	205 (95.3%)	0.130
Yes	34 (2.7%)	10 (4.7%)	
Chronic respiratory disease			
No	989 (80.0%)	166 (77.2%)	0.250
Asthma	76 (6.1%)	8 (3.7%)	
COPD	77 (6.2%)	19 (8.8%)	
ILD	4 (0.3%)	1 (0.5%)	
Other	91 (7.4%)	21 (9.8%)	
Radiologic findings			
Bilateral infiltrates	Missing N = 90	Missing N = 37	
No	524 (45.7%)	63 (35.4%)	0.010
Yes	623 (54.3%)	115 (64.6%)	
Severity			
SpO_2_/FiO_2_ ratio at admission	452.38 (438.10–461.90)	433.33 (370.83–457.14)	< 0.001
CURB65 score	1 (0–2)	2 (1–3)	< 0.001
Biochemical parameters at admission			
Urea, mg/dL	32 (25–43)	51 (38–71)	< 0.001
C-reactive protein, mg/L	65.20 (29.85–123.54)	117.08 (63.80–193.64)	< 0.001
D-dimer, ng/mL	700 (404–1200)	1200 (720–2300)	< 0.001
Leucocyte count, cells/mL	6200 (4690–8210)	6500 (4930–9660)	0.046
Neutrophil count, cells/mL	4620 (3300–6400)	5100 (3550–8100)	0.005
Lymphocyte count, cells/mL	910 (690–1270)	680 (500–1040)	< 0.001
Platelet count, 10^3^ cells/mL	187 (146–242)	172.5 (121–220)	< 0.001

Data are summarized as n (%) or median (interquartile range). Abnormal markers: lymphocyte count < 724 cells/mm^3^, CRP > 60 mg/L, SpO_2_/FiO_2_ < 450. *COPD* chronic obstructive pulmonary disease; *ILD* interstitial lung disease; *IQR* interquartile range; *SpO*_*2*_*/FiO*_*2*_ peripheral blood oxygen saturation/fraction of inspired oxygen. Continuous variables are compared using the Wilcoxon rank-sum and categorical variables are compared using Pearson's chi-squared test.

A total of 1292 patients were included in the COVID-19 2020 validation cohort. Of these, 1222 had complete data for the selected variables (Supplementary Fig. 1). Supplementary Table 2 shows baseline characteristics of the validation cohort. Moreover, baseline characteristics according to the number of markers (ALC, CRP, and SpO_2_/FiO_2_) altered can be found in supplementary Table 3. A total of 462 patients were included in the COVID-19 2022–23 validation cohort.

A total of 1601 patients were included in the CAP cohort. Of these, 1292 had complete data for the selected variables and outcome under study (Supplementary Fig. 2). Supplementary Table 4 shows baseline characteristics according to in-hospital mortality. In addition, supplementary Table 5 shows baseline characteristics according to the number of markers (ALC, CRP, and SpO_2_/FiO_2_) altered.

### Primary outcome: in-hospital mortality

**Figure 2:**
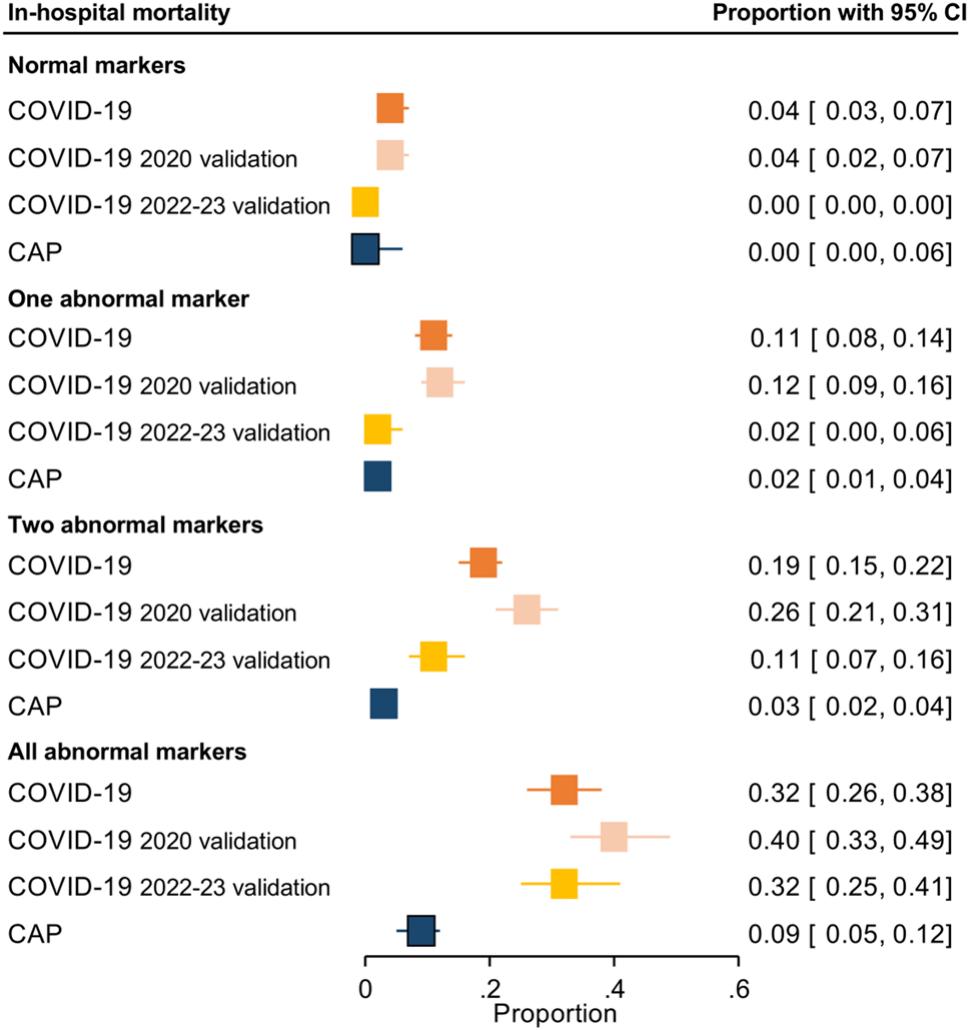
Proportion of in-hospital mortality according to abnormal markers. *CAP* community-acquired pneumonia; *CI* confidence interval.

**Figure 3:**
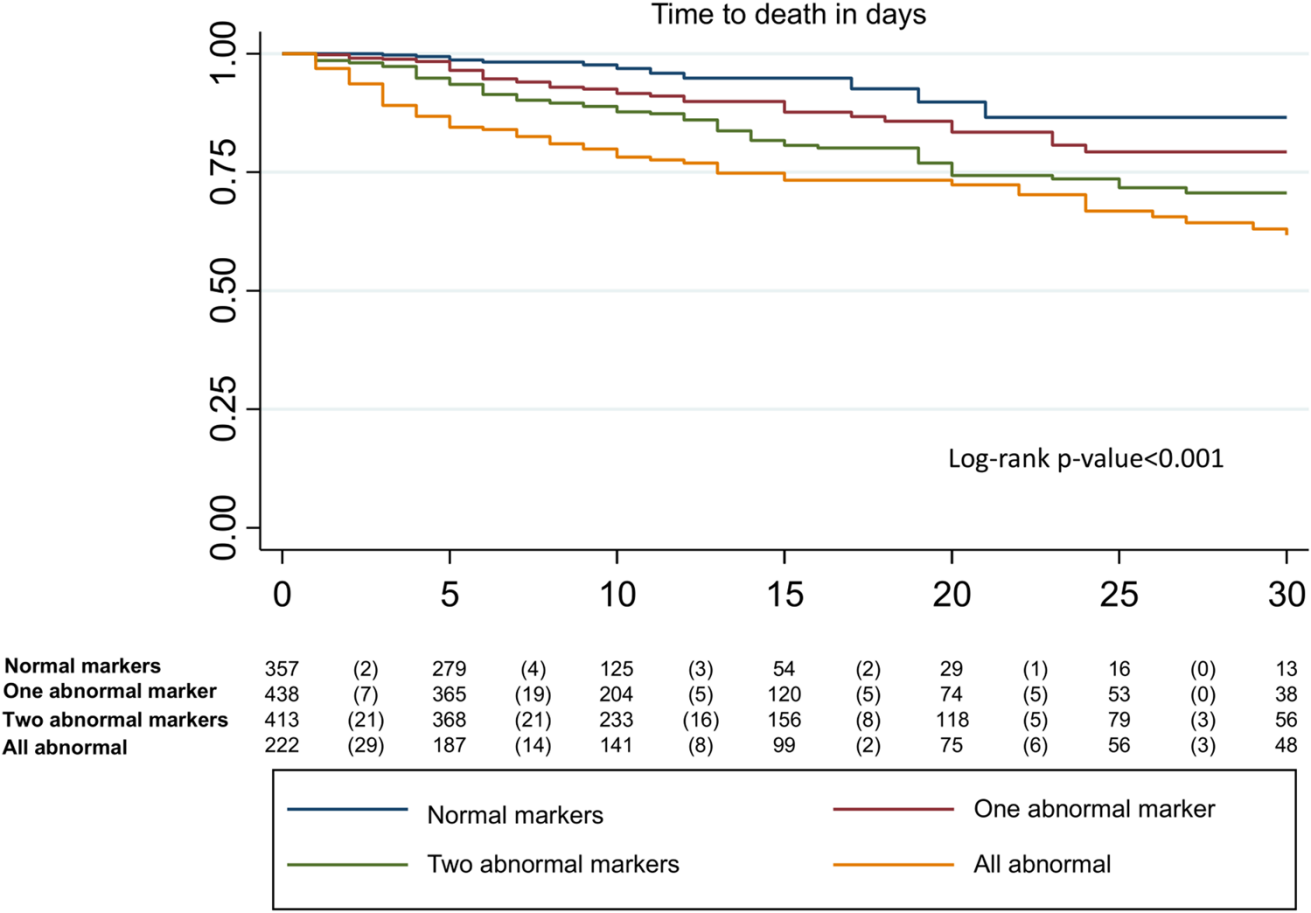
Time to in-hospital mortality according to abnormal markers.

In-hospital mortality was 14.8% (215 of 1452), 17.1% (221 of 1292), 13.6% (63 of 462), and 3.6% (47 of 1292) for the three COVID-19 (derivation, validation 2020 and 2022–23 validation cohorts, respectively) and CAP cohorts, respectively. Depending on whether all markers were normal or altered, mortality ranged from 4–32%, 4–40%, 0–32%, and 0–9% in the COVID-19, COVID-19 2020 validation, COVID-19 2022–23 validation and CAP cohorts, respectively (Table 2 and Fig. 2). The progressive increase in odds for in-hospital mortality in COVID-19 per the number of biomarkers altered was confirmed after we adjusted for age and sex (Table 2). The area under the receiver operating curve (AUROC) was 0.83 (0.80;0.86) and 0.83 (0.80;0.86) for the COVID-19 and the COVID-19 validation 2020 cohorts, respectively. Again, adding CURB65 to the model, similar results were found after adjusting for age and sex (supplementary Table 6). This analysis with CURB65 could not be performed in the validation COVID-19 cohorts due to the high number of missing data. To assess the influence of time since symptom onset, a subgroup analysis was performed according to whether the patients had had symptoms for less than 7 days or not (Table 3). This analysis showed a similar increase in odds of death according to the number of markers altered. The COVID-19 and CAP cohorts presented a comparable trend, with the latter presenting an AUROC of 0.75 (0.68;0.82) (Table 2). However, due to the low number of events, CIs were very wide and results were not statistically significant. As shown in Fig. 3, the number of altered markers allows various groups to already be differentiated within the first days per the risk of mortality.

**Table 2:** COVID-19 and CAP data association tables for in-hospital mortality and penalized logistic regression analysis.

Biomarkers	Events	N	% Deaths (CI 95%)	*OR (95% CI)
COVID-19 cohort				
Normal markers	15	358	4.2% (2.5; 6.8)	–
One abnormal marker	47	440	10.7% (8.1; 13.9)	1.82 (0.97; 3.41)
Two abnormal markers	78	419	18.6% (15.2; 22.6)	3.03 (1.67; 5.52)
All abnormal markers	75	235	31.9% (26.3; 38.2)	6.30 (3.39; 11.69)
Total	215	1452	14.8% (13.1; 16.7)	–
COVID-19 2020 validation cohort				
Normal markers	12	314	3.82% (2.2; 6.6)	–
One abnormal marker	53	437	12.13% (9.4; 15.5)	2.65 (1.37;5.11)
Two abnormal markers	84	327	25.69% (21.2; 30.7)	6.10 (3.20;11.63)
All abnormal markers	58	144	40.28% (32.6; 48.5)	9.80 (4.93;19.49)
Total	207	1222	16.94% (14.9; 19.1)	–
COVID-19 2022–23 validation cohort				
Normal markers	0	0	–	–
One abnormal marker	2	132	1.5% (0.4; 5.9)	–
Two abnormal markers	23	213	10.8% (7.2; 15.7)	4.25 (1.12; 16.21)
All abnormal markers	38	117	32.5% (24.6; 41.5)	16.03 (4.26; 60.32)
Total	63	462	13.6% (10.8; 17.1)	–
CAP cohort				
Normal markers	0	57	0.0% (0.0; 6.2)	–
One abnormal marker	9	404	2.2% (1.0; 4.2)	2.85 (0.16; 49.99)
Two abnormal markers	17	588	2.9% (1.7; 4.6)	2.95 (0.17; 50.11)
All abnormal markers	21	243	8.6% (5.4; 12.9)	8.74 (0.52; 147.60)
Total	47	1292	3.6% (2.7; 4.8)	–

Penalized logistic regression model performed.

*CAP* community-acquired pneumonia; *CI* confidence interval; *OR* odds ratio.

*Adjusted by sex and age.

**Table 3:** Logistic regression analysis for in-hospital mortality and ICU admission in COVID-19 according to time since symptom onset.

	COVID-19 cohort		CAP cohort	
	< 7 days	≥ 7 days	< 7 days	≥ 7 days
Abnormal biomarkers	OR (95% CI)	OR (95% CI)	OR (95% CI)	OR (95% CI)
In-hospital mortality				
Normal markers	–	–	–	–
One abnormal marker	1.4 (0.7;3.1)	2.6 (0.9;7.7)	1.9 (0.1;35.8)	1.0 (0.1;20.7)
Two abnormal markers	2.2 (1.1;4.7)	4.9 (1.7;13.7)	1.8 (0.1;32.2)	1.2 (0.1;22.1)
All abnormal markers	3.5 (1.6;8.0)	12.6 (4.5;35.6)	5.8 (0.3;100.7)	2.9 (0.2;55.7)
AUROC	0.80 (0.76;0.84)	0.86 (0.82;0.89)	0.72 (0.63;0.81)	0.81 (0.72;0.89)
ICU admission				
Normal markers	–	–	–	–
One abnormal marker	2.4 (1.2;5.1)	5.9 (2.6;13.3)	1.6 (0.1;30.2)	0.8 (0.0;16.8)
Two abnormal markers	6.1 (2.9;12.6)	15.3 (6.8;34.4)	6.3 (0.4;106.1)	3.3 (0.2;59.7)
All abnormal markers	9.0 (4.0;20.2)	33.2 (14.2;77.6)	20.4 (1.2;347.3)	12.6 (0.7;226.3)
AUROC	0.74 (0.69;0.79)	0.78 (0.74;0.81)	0.75 (0.69;0.81)	0.78 (0.67;0.88)

*AUROC* area under the receiver operating point; *CAP* community-acquired pneumonia; *CI* confidence interval; *ICU* intensive care unit; *OR* odds ratio. Normal markers as reference group.

*Age and sex adjusted.

**Figure 4:**
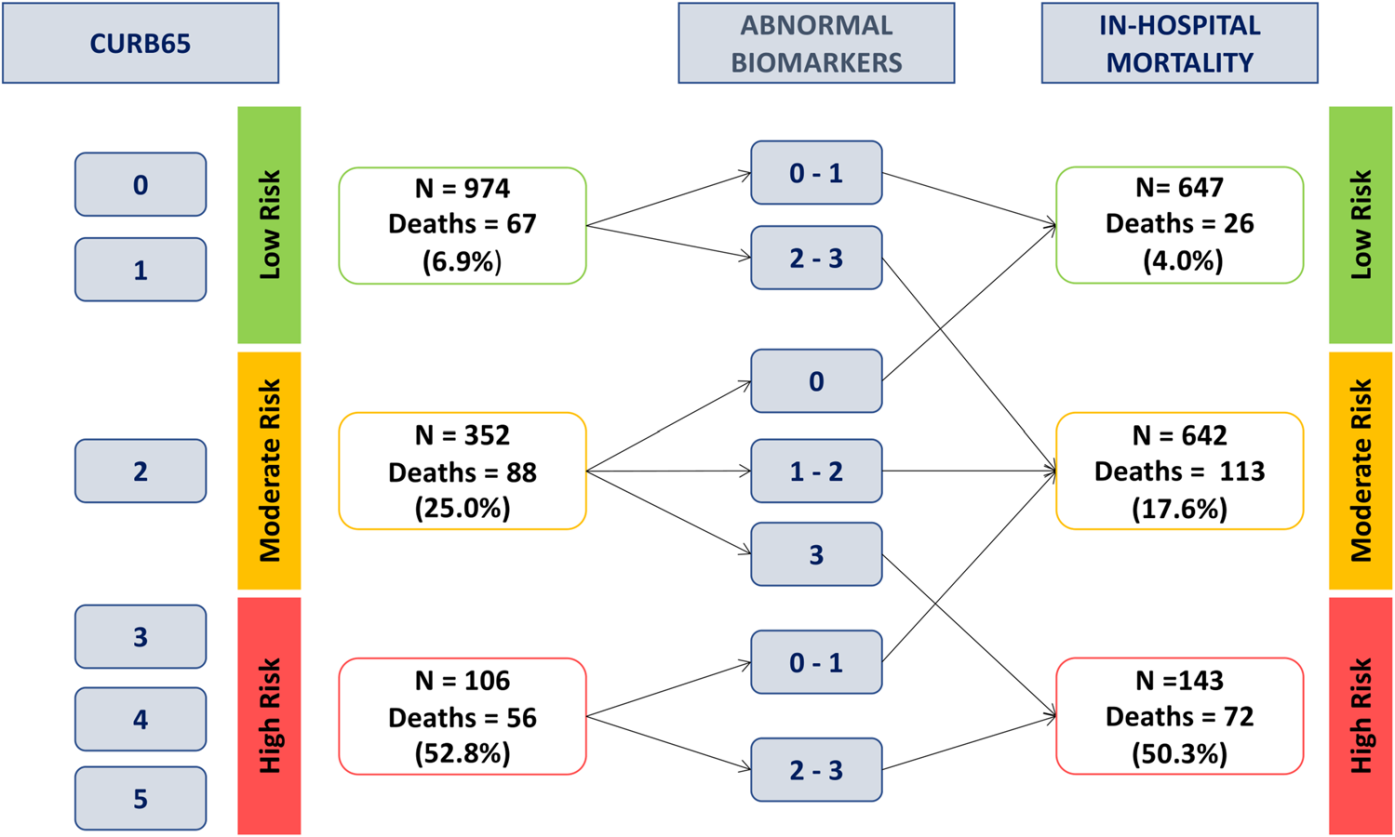
Net reclassification analysis (NRI) for the COVID-19 derivation cohort. The effect of combining the CURB65 score with the 3 biomarkers model for in-hospital mortality is shown.

A net reclassification analysis (NRI) for the COVID-19 derivation cohort was calculated to evaluate the effect of combining the CURB65 score with the 3 biomarkers model (Fig. 4).

### Secondary outcome: ICU admission

**Figure 5:**
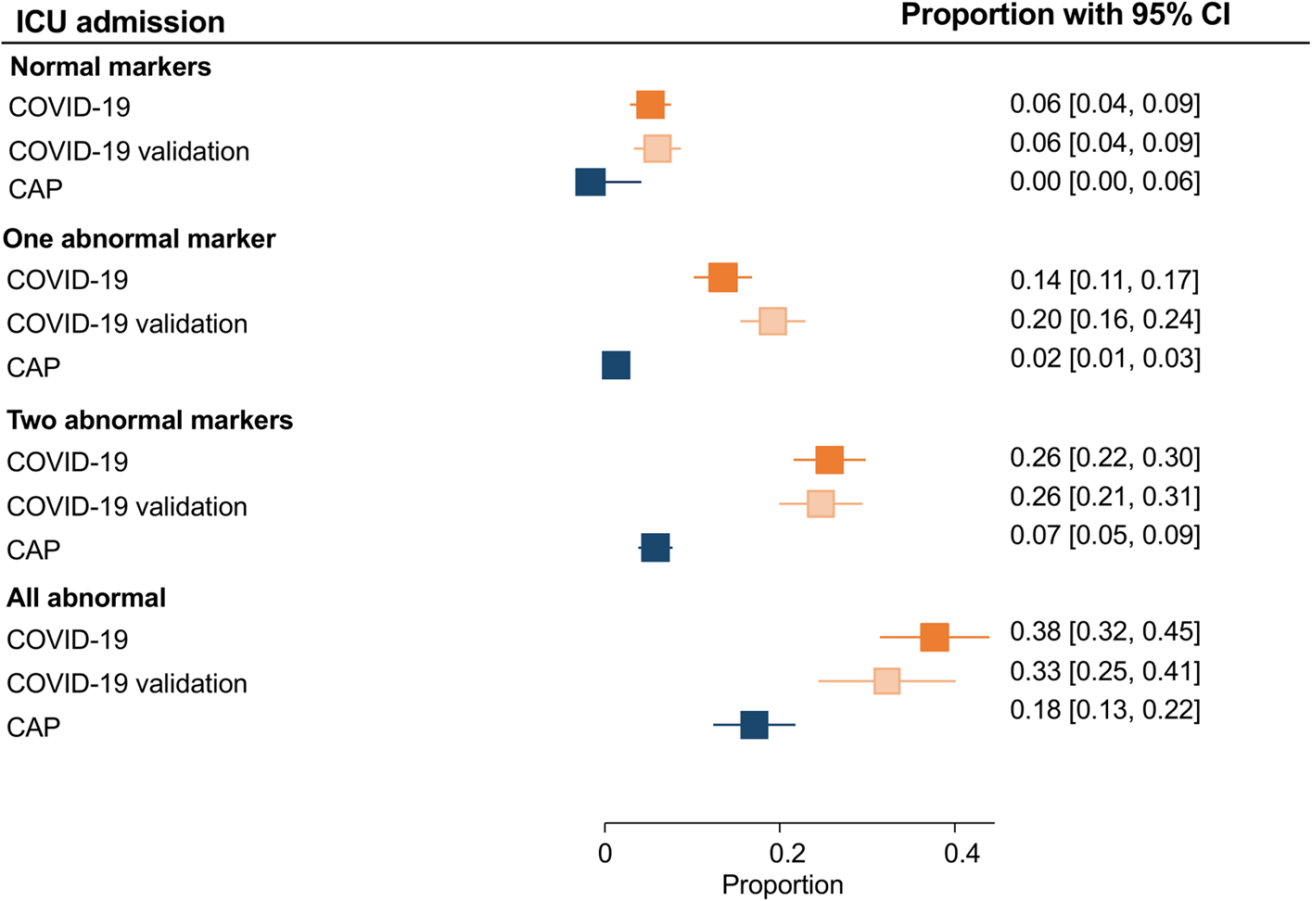
Proportion of ICU admission according to abnormal markers. *CAP* community-acquired pneumonia; *CI* confidence interval; *ICU* intensive care unit.

**Figure 6:**
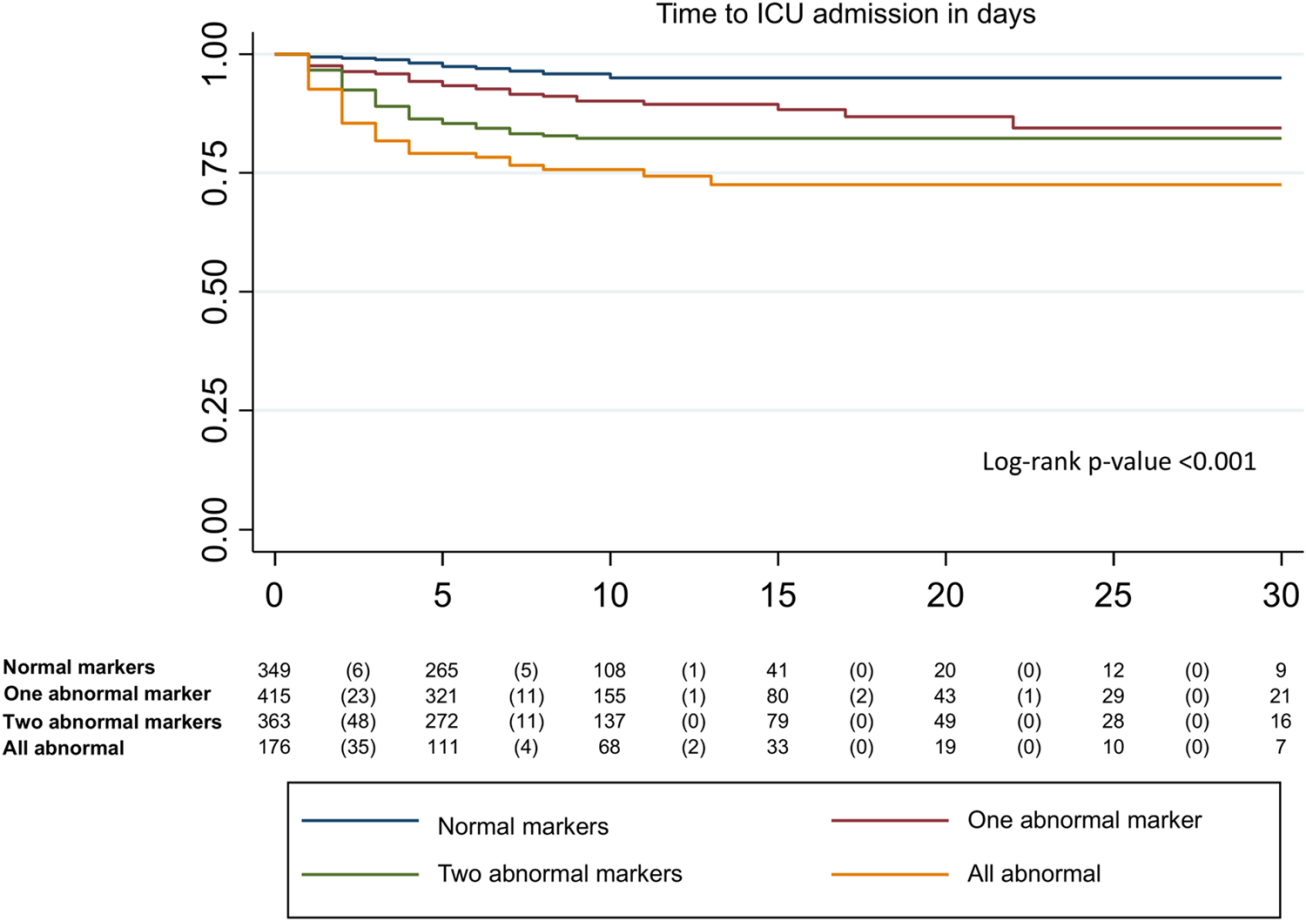
Time to ICU admission according to abnormal markers. *ICU* intensive care unit.

ICU admission was necessary for 282 (19.4%), 232 (16.9%) and 89 (6.9%) patients in the COVID-19, COVID-19 validation and CAP cohorts, respectively. The proportion of ICU admissions rose in both cohorts as the number of altered markers grew (Table 4 and Fig. 5). After adjusting for age and sex, we observed that involvement of the immune, inflammatory and respiratory responses was related to increased odds for ICU admission in COVID-19 (Table 3). The AUROC was 0.76 (0.73;0.79) and 0.76 (0.73;0.79) for the COVID-19 and COVID-19 validation cohorts. Table 3 shows the logistic regression analysis for ICU admission with similar findings according to time since symptom onset. In the CAP cohort, we found a comparable trend for ICU admission to that of the COVID-19 cohort; AUROC was 0.76 (0.71;0.82) (Table 3). However, due to the low number of events, the CIs were very wide, and the results were not statistically significant. As with mortality, the number of altered markers differentiated the groups within the first days, according to the risk of ICU admission (Fig. 6).

**Table 4:** COVID-19 and CAP data association tables for ICU admission and penalized logistic regression analysis.

Biomarkers	Events	N	% ICU admissions (CI 95%)	*OR (95% CI)
COVID-19 cohort				
Normal markers	20	358	6.0% (3.6; 8.5)	–
One abnormal marker	62	440	14.1% (11.1; 17.6)	3.65 (2.14;6.25)
Two abnormal markers	110	419	26.3% (22.3; 30.7)	9.53 (5.60;16.2)
All abnormal markers	90	235	38.3% (32.3; 44.7)	18.16 (10.32;31.95)
Total	282	1452	19.4% (17.5; 21.5)	–
COVID-19 2020 validation cohort				
Normal markers	20	314	6.4% (4.1;9.7)	–
One abnormal marker	86	435	19.8% (16.3;23.8)	3.44 (2.06;5.73)
Two abnormal markers	81	320	25.3% (20.8;30.4)	4.63 (2.74;7.81)
All abnormal markers	45	137	32.8% (25.5;41.1)	6.62 (3.68;11.91)
Total	232	1206	19.2% (17.1;21.6)	–
CAP cohort				
Normal markers	0	57	0.0% (0.0; 6.2)	–
One abnormal marker	8	404	2.0% (0.9; 4.0)	2.27 (0.13;40.26)
Two abnormal markers	38	588	6.5% (4.7; 8.7)	9.71 (0.59;161.22)
All abnormal markers	43	243	17.7% (13.4; 23.0)	33.61 (2.02;559.47)
Total	89	1292	7% (5.6; 8.4)	–

Penalized logistic regression model performed and adjusted by sex and age.

*CI* confidence interval; *ICU* intensive care unit; *OR* odds ratio.

*Adjusted by sex and age.

## Discussion

The main findings of the study were as follows: (i) three feasible-to-obtain parameters at pneumonia diagnosis –CRP > 60 mg/L, lymphocyte count < 724 cells/mm^3^ and, SpO_2_/FiO_2_ < 450– were able to stratify the risk of mortality into four groups; (ii) the mortality rate ranged between 4 and 32% (from 0 to 3 parameters altered) in COVID-19 and 0–9% in CAP; (iii) that simple tool showed usefulness in two different microbiological pneumonia scenario: COVID-19 and CAP although with worse performance in CAP.From the start of the COVID-19 pandemic and like in CAP, the search for useful prognostic scales in routine practice has generated great interest. Many studies have received wide acceptance whilst others have presented limitations^14^. Multiple scales and scores have shown their usefulness for short- and long-term mortality risk. However, none of them address the host's response with basic parameters, measured in emergency rooms, in such a simple and practical way and this represents a different personalized approach. Our study explored a basic and three-dimensional approach to host response that included performing a blood analysis that provided lymphocyte count and CRP (surrogate of inflammation) and measuring oxygen saturation (surrogate of lung function). This approach allowed for patient stratification into four subgroups of increasing severity similar to other strategies in different conditions and settings^15^. This information offers additional utility to well-known and wide used scores as CURB65 to discern risk more accurately because if the three biomarkers are abnormal, mortality increases despite a low CURB65 punctuation and vice versa. We selected easy parameters available in all hospitals and many facilities, and attained thresholds previously published in other studies. In CAP, a lymphopenia < 724 cells/mm^3^ had been reported to be associated with increased mortality after adjusting for CURB65 scale whilst, in COVID-19, lymphopenia was a constant finding in the most severe episodes^7,8,16^. If an infection is present, lymphopenia could indicate poor control of the pathogen. For example, it may lead to persistent hypercytokinemia in cases of CAP^8^. Although several biomarkers have been related to outcomes in COVID-19 and CAP^17–19^, CRP has been used in clinical scores and trials exploring the use of immunomodulators^5,20,21^. Similarly, oxygenation level—representing the main physiological function of the lung—was computed through SpO_2_/FiO_2_ ratio. It corresponded to an SpO_2_ 94% at room air and which was widely used in many publications and trials for COVID-19^22^.

Our approach is easy to implement because it depends on only three parameters. The alteration of a single factor–inflammation, lymphocyte count or oxygenation level–approximately doubles the odds of death. When all three factors are altered, there is a more-than-sixfold increase in such risk. This information is valuable, as it can influence resource optimization, patient allocation and mortality risk calculations. Adjusted for age and sex, our analysis had an AUROC of 0.83 for the mortality risk. This is similar to the most widespread, and complex, COVID-19-specific scores: the 4C Mortality Score (8 items) with an AUROC of 0.79 for in-hospital mortality and the ISARIC 4C Deterioration model (11 items) for clinical deterioration^5,23^. CAP scores evaluated for COVID-19 showed AUROC of less than 0.8 in all cases as well^24^.

As time course is an important aspect in pathogenesis–the initial viral replication and inflammation phases–to consider for stratifying risk, our model was probed in both stages. It performed similarly within the first seven days (AUROC 0.74 [0.69;0.79]) since symptom onset and thereafter (AUROC 0.78 [0.74;0.81]). Our multidimensional approach was reproduced in both a different cohort of SARS-CoV-2 pneumonia and in patients with CAP.

There is no certainty about how new variants would modify the epidemiology of CAP and COVID-19, though SARS-CoV-2 became an endemic virus, like other coronaviruses (HCoV-229E, HCoV-OC43, HCoV-NL63, and HCoV-HKU1)^25^. In this new scenario, clinicians will have to face pneumonia of different or combined etiologies, including SARS-CoV-2, other viruses, and common bacteria. Basic tools will be necessary to assess and characterize clinical profiles of host response, irrespective of microbiologic etiology. Our present study aimed to address this aspect. The need for such an intervention reinforces the use of the model application and its extensibility to any pneumonia. The strength of our approach is its simplicity in quickly and pragmatically identifying patients with higher risk of worse outcomes, especially in the absence of sophisticated studies in some hospitals or other facilities. Other advantages include the use of objective data and adjustments in our analysis by age and sex.

The study has several limitations, though. The COVID-19 cohort had an in-hospital mortality four times higher than that of the CAP cohort. Nonetheless, the model behaved identically when evaluating the host response; there was an increase in risk per the number of markers in both cohorts. The COVID-19 cohort was recruited when there were no specific treatments. The COVID cohorts were established in the early phase of the pandemic when the healthcare system was overloaded. The scarcity of health care resources may introduce bias into the analysis. The potential impact of vaccination with induced immunity and the appearance of variants with varying virulence may have affected the course and outcomes of the disease. It is, therefore, necessary to confirm these data in the future. The strengths of our study are multi-site cohorts and the evaluation of interplay between microorganism and host by the approach. This latter detail is important, as it allows personalized risk assessment. Quoting DeMerle et al.^1^, “*not all hosts nor all host responses are the same*”. A step towards more personalized medicine in COVID-19 and CAP does not always imply the necessary use of complex techniques. CRP, ALC, and SpO_2_/FiO_2_ are routinely available at both low- and high-resource settings.

In conclusion, we presented a feasible and streamlined multidimensional approach to assess pneumonia severity per the host response and regardless of age or sex. Three simple biomarkers mirroring inflammatory, immune, and respiratory function responses allow for an easy on-site or point-of-care strategy that facilitates severity assessment in varying hospitals. In the future, tools capable of identifying different severity profiles based on the characteristics of the host will be necessary to improve overall care and optimize patient and resource allocation.

### Supplementary Information

Supplementary Information.

## Data Availability

The data that support the findings of this study are available from the corresponding author, RM, upon reasonable request.
