# Supplementary material for: Basic host response parameters to classify mortality risk in COVID-19 and community-acquired pneumonia
Source: Sci Rep. 2024 Jun 3;14:12726. doi: 10.1038/s41598-024-62718-4 (PMC11148180; doi:10.1038/s41598-024-62718-4)
Supplement: Supplementary file 1 — Supplementary Information. [file 41598_2024_62718_MOESM1_ESM.docx]

**Supplemental file**

**Basic host response parameters to classify mortality risk in COVID-19 and community-acquired pneumonia**

Supplementary Table 1. Baseline characteristics of COVID-19 cohort according to abnormal markers.

| **Variables** | **Normal markers n=358** | **One abnormal marker n=440** | **Two abnormal markers n=419** | **All abnormal markers n=235** | **P-value** |
| --- | --- | --- | --- | --- | --- |
| **Demographics** |  |  |  |  |  |
| Age (IQR) | 55.5 (43.00-70.00) | 64.0 (52.00-76.00) | 69.0 (58.00-79.00) | 71.0 (62.00-79.00) | <0.001 |
| Sex, no. (%) |  |  |  |  |  |
| Female | 176 (49.2%) | 182 (41.4%) | 148 (35.3%) | 65 (27.7%) | <0.001 |
| Male | 182 (50.8%) | 258 (58.6%) | 271 (64.7%) | 170 (72.3%) | 0.54 |
| Smoking, no. (%) | 237 (68.9%) | 280 (69.5%) | 241 (66.2%) | 127 (65.5%) |  |
| No smokers | 26 (7.6%) | 25 (6.2%) | 20 (5.5%) | 10 (5.2%) | 0.54 |
| Current smokers | 81 (23.5%) | 98 (24.3%) | 103 (28.3%) | 57 (29.4%) |  |
| Ex-smokers | 237 (68.9%) | 280 (69.5%) | 241 (66.2%) | 127 (65.5%) |  |
| **Comorbidities** |  |  |  |  |  |
| Arterial hypertension, no. (%) |  |  |  |  |  |
| No | 236 (65.9%) | 227 (51.6%) | 170 (40.6%) | 89 (37.9%) | <0.001 |
| Yes | 122 (34.1%) | 213 (48.4%) | 249 (59.4%) | 146 (62.1%) |  |
| Diabetes, no. (%) |  |  |  |  |  |
| No | 316 (88.5%) | 354 (80.6%) | 308 (73.5%) | 166 (70.9%) | <0.001 |
| Yes | 41 (11.5%) | 85 (19.4%) | 111 (26.5%) | 68 (29.1%) |  |
| Dyslipidemia, no. (%) |  |  |  |  |  |
| No | 253 (70.7%) | 275 (62.5%) | 237 (56.6%) | 144 (61.3%) | <0.001 |
| Yes | 105 (29.3%) | 165 (37.5%) | 182 (43.4%) | 91 (38.7%) |  |
| Chronic heart disease, no. (%) |  |  |  |  |  |
| No | 318 (88.8%) | 362 (82.3%) | 326 (77.8%) | 180 (76.6%) | <0.001 |
| Yes | 40 (11.2%) | 78 (17.7%) | 93 (22.2%) | 55 (23.4%) |  |
| Chronic renal disease, no. (%) |  |  |  |  |  |
| No | 270 (92.5%) | 286 (88.3%) | 246 (82.3%) | 168 (89.4%) | 0.002 |
| Yes | 22 (7.5%) | 38 (11.7%) | 53 (17.7%) | 20 (10.6%) |  |
| Chronic liver disease, no. (%) |  |  |  |  |  |
| No | 348 (97.2%) | 429 (97.5%) | 403 (96.2%) | 228 (97.0%) | 0.71 |
| Yes | 10 (2.8%) | 11 (2.5%) | 16 (3.8%) | 7 (3.0%) |  |
| Chronic respiratory disease, no. (%) |  |  |  |  |  |
| No | 294 (82.1%) | 356 (80.9%) | 322 (76.8%) | 183 (77.9%) | 0.018 |
| Asthma | 29 (8.1%) | 25 (5.7%) | 22 (5.3%) | 8 (3.4%) |  |
| COPD | 14 (3.9%) | 24 (5.5%) | 37 (8.8%) | 21 (8.9%) |  |
| ILD | 2 (0.6%) | 0 (0.0%) | 1 (0.2%) | 2 (0.9%) |  |
| Other | 19 (5.3%) | 35 (8.0%) | 37 (8.8%) | 21 (8.9%) |  |
| **Radiologic findings** |  |  |  |  |  |
| Bilateral infiltrates, no. (%) |  |  |  |  |  |
| No | 200 (57.1%) | 199 (48.9%) | 131 (34.7%) | 57 (29.8%) | <0.001 |
| Yes | 150 (42.9%) | 208 (51.1%) | 246 (65.3%) | 134 (70.2%) |  |
| **Severity** |  |  |  |  |  |
| SpO2/FiO2 ratio at admission (IQR) | 461.90 (457.14-466.67) | 457.14 (447.62-461.90) | 442.86 (414.29-452.38) | 419.05 (347.62-438.10) | <0.001 |
| CURB65 score (IQR) | 0 (0-1) | 1 (0-2) | 1 (1-2) | 1 (1-2) | <0.001 |
| **Biochemical parameters at admission** | |  |  |  |  |
| Urea, mg/dL (IQR) | 29 (23-36) | 33 (25-48) | 39 (29-56) | 41 (31-55) | <0.001 |
| C-reactive protein, mg/L (IQR) | 24.63 (10.10-39.30) | 61.38 (32.22-106.03) | 111.20 (75.18-160.34) | 156.30 (108.90-220) | <0.001 |
| D-dimer, ng/mL (IQR) | 494 (300-844) | 700 (430-1260) | 900 (561.50-1585) | 1000 (643-1832) | <0.001 |
| Leucocyte count, cells/mL (IQR) | 5730 (4550-7090) | 6100 (4600-8110) | 6780 (4890-9500) | 6900 (5090-9170) | <0.001 |
| Neutrophil count, cells/mL (IQR) | 3910 (3000-5050) | 4405 (3155-6110) | 5280 (3600-7570) | 5900 (4150-8100) | <0.001 |
| Lymphocyte count, cells/mL (IQR) | 1200 (930-1550) | 980 (760-1300) | 800 (600-1110) | 520 (400-620) | <0.001 |
| Platelet count, 10^3^ cells/mL (IQR) | 188 (148-239) | 184 (143-236) | 182 (141-244.5) | 191 (144.5-236) | 0.66 |

Data are summarized as n (%) or median (interquartile range). Abnormal markers: lymphocyte count < 724 cells/mm^3^, CRP > 60 mg/L, SpO2/FiO2 < 450. Abbreviations: COPD, chronic obstructive pulmonary disease; ILD, interstitial lung disease; IQR, interquartile range; SpO2/FiO2, peripheral blood oxygen saturation/fraction of inspired oxygen. Continuous variables are compared using the Kruskal-Wallis test and categorical variables are compared using Pearson's chi-squared test.

Supplementary Table 2. Baseline characteristics of COVID-19 validation cohort according to in-hospital mortality.

| **Variables** | **Alive**  **n=1015** | **Deceased**  **n=207** | **P-value** |
| --- | --- | --- | --- |
| **Demographics** |  |  |  |
| Age | 68 (58-76) | 82 (74-87) | <0.001 |
| Sex |  |  |  |
| Female | 454 (44.7%) | 79 (38.2%) | 0.083 |
| Male | 561 (55.3%) | 128 (61.8%) |  |
| Smoking | Missing N=269 | Missing N=10 |  |
| No smokers | 511 (68.5%) | 137 (69.5%) | 0.780 |
| smokers | 235 (31.5%) | 60 (30.5%) |  |
| **Comorbidities** |  |  |  |
| Arterial hypertension |  |  |  |
| No | 539 (53.1%) | 70 (33.8%) | <0.001 |
| Yes | 476 (46.9%) | 137 (66.2%) |  |
| Diabetes |  |  |  |
| No | 843 (83.1%) | 132 (63.8%) | <0.001 |
| Yes | 172 (16.9%) | 75 (36.2%) |  |
| Dyslipidemia | Missing N=4 | Missing N=1 |  |
| No | 648 (64.1%) | 116 (56.3%) | 0.035 |
| Yes | 363 (35.9%) | 90 (43.7%) |  |
| Chronic renal disease | Missing N=302 | Missing N=47 |  |
| No | 960 (94.6%) | 166 (80.2%) | <0.001 |
| Yes | 55 (5.4%) | 41 (19.8%) |  |
| Chronic liver disease |  |  |  |
| No | 980 (96.6%) | 199 (96.1%) | 0.770 |
| Yes | 35 (3.4%) | 8 (3.9%) |  |
| **Severity** |  |  |  |
| SpO2/FiO2 ratio at admission | 452 (438-457) | 428 (371-447) | <0.001 |
| **Biochemical parameters at admission** |  |  |  |
| Urea, mg/dL | 37 (29-48) | 64 (48-103) | <0.001 |
| C-reactive protein, mg/L | 25.2 (7.4-80.0) | 89.2 (26.2-151.4) | <0.001 |
| D-dimer, ng/mL | 657 (423-1111) | 1170.50 (692-2028) | <0.001 |
| Leucocyte count, cells/mL | 5505 (4290-7400) | 6450 (4950-9550) | <0.001 |
| Neutrophil count, cells/mL | 3900 (2860-5710) | 5515 (3730-8040) | <0.001 |
| Lymphocyte count, cells/mL | 930 (690-1290) | 700 (460-1000) | <0.001 |
| Platelet count, 10^3^ cells/mL | 178 (138-233) | 169 (126-222) | 0.035 |

Data are summarized as n (%) or median (interquartile range). Abnormal markers: lymphocyte count < 724 cells/mm^3^, CRP > 60 mg/L, SpO2/FiO2 < 450. Abbreviations: COPD, chronic obstructive pulmonary disease; ILD, interstitial lung disease; IQR, interquartile range; SpO2/FiO2, peripheral blood oxygen saturation/fraction of inspired oxygen. Continuous variables are compared using the Wilcoxon rank-sum and categorical variables are compared using Pearson's chi-squared test.

Supplementary Table 3. Baseline characteristics of COVID-19 cohort validation according to abnormal markers.

| **Variables** | **Normal markers n=315** | **One abnormal marker n=438** | **Two abnormal markers n=329** | **All abnormal markers n=144** | **P-value** |
| --- | --- | --- | --- | --- | --- |
| **Demographics** |  |  |  |  |  |
| Age (IQR) | 64 (51-75) | 70 (60-79) | 72 (62-81) | 77 (69-84) | <0.001 |
| Sex, no. (%) |  |  |  |  |  |
| Female | 170 (54.0%) | 196 (44.7%) | 116 (35.3%) | 53 (36.8%) | <0.001 |
| Male | 145 (46.0%) | 242 (55.3%) | 213 (64.7%) | 91 (63.2%) |  |
| Smoking, no. (%) |  |  |  |  |  |
| No smokers | 161 (72.5%) | 230 (67.1%) | 175 (67.0%) | 85 (70.2%) | 0.490 |
| Smokers | 61 (27.5%) | 113 (32.9%) | 86 (33.0%) | 36 (29.8%) |  |
| **Comorbidities** |  |  |  |  |  |
| Arterial hypertension, no. (%) |  |  |  |  |  |
| No | 178 (56.5%) | 223 (50.9%) | 149 (45.3%) | 60 (41.7%) | 0.006 |
| Yes | 137 (43.5%) | 215 (49.1%) | 180 (54.7%) | 84 (58.3%) |  |
| Diabetes, no. (%) |  |  |  |  |  |
| No | 269 (85.4%) | 356 (81.3%) | 245 (74.5%) | 108 (75.0%) | 0.002 |
| Yes | 46 (14.6%) | 82 (18.7%) | 84 (25.5%) | 36 (25.0%) |  |
| Dyslipidemia, no. (%) |  |  |  |  |  |
| No | 202 (64.3%) | 275 (63.1%) | 202 (61.6%) | 88 (61.5%) | 0.890 |
| Yes | 112 (35.7%) | 161 (36.9%) | 126 (38.4%) | 55 (38.5%) |  |
| Chronic renal disease, no. (%) |  |  |  |  |  |
| No | 297 (94.3%) | 410 (93.6%) | 291 (88.4%) | 132 (91.7%) | 0.023 |
| Yes | 18 (5.7%) | 28 (6.4%) | 38 (11.6%) | 12 (8.3%) |  |
| Chronic liver disease, no. (%) |  |  |  |  |  |
| No | 308 (97.8%) | 415 (94.7%) | 319 (97.0%) | 140 (97.2%) | 0.120 |
| Yes | 7 (2.2%) | 23 (5.3%) | 10 (3.0%) | 4 (2.8%) |  |
| Yes |  |  |  |  |  |
| **Severity** |  |  |  |  |  |
| SpO2/FiO2 ratio at admission (IQR) | 462 (452-467) | 448 (438-457) | 438 (414-447) | 423 (383-438) | <0.001 |
| **Biochemical parameters at admission** | |  |  |  |  |
| Urea, mg/dL (IQR) | 35 (27-43) | 39 (30-52) | 45 (33-68) | 49.50 (38-72) | <0.001 |
| C-reactive protein, mg/L (IQR) | 10.2 (4.0-26.7) | 18.4 (6.4-49.4) | 90.8 (31.7-142.6) | 139.80 (98.7-209.8) | <0.001 |
| D-dimer, ng/mL (IQR) | 548 (380-970) | 659.50 (418-1072) | 846 (531-1446) | 1077 (595-1868) | <0.001 |
| Leucocyte count, cells/mL (IQR) | 5220 (4320-6730) | 5480 (4280-7460) | 6090 (4560-8520) | 6395 (4525-8265) | <0.001 |
| Neutrophil count, cells/mL (IQR) | 3500 (2600-4640) | 3870 (2900-5790) | 4750 (3230-7080) | 5610 (3775-7320) | <0.001 |
| Lymphocyte count, cells/mL (IQR) | 1150 (970-1500) | 945 (700-1340) | 740 (560-1000) | 510 (400-620) | <0.001 |
| Platelet count, 10^3^ cells/mL (IQR) | 179000 (138-237) | 173 (136-225) | 172 (134-228) | 189 (148-247) | 0.280 |
| Data are summarized as n (%) or median (interquartile range). Abnormal markers: lymphocyte count < 724 cells/mm^3^, CRP > 60 mg/L, SpO2/FiO2 < 450. Abbreviations: COPD, chronic obstructive pulmonary disease; ILD, interstitial lung disease; IQR, interquartile range; SpO2/FiO2, peripheral blood oxygen saturation/fraction of inspired oxygen. Continuous variables are compared using the Kruskal-Wallis test and categorical variables are compared using Pearson's chi-squared test. | | | | | |

Supplementary Table 4. Baseline characteristics of CAP cohort according to in-hospital mortality.

| **Variables** | **Alive**  **n=1245** | **Deceased**  **n=47** | **P-value** |
| --- | --- | --- | --- |
| **Demographics** |  |  |  |
| Age (IQR) | 72 (58-80) | 82 (72-88) | <0.01 |
| Sex, no. (%) |  |  |  |
| Female | 471 (37.8%) | 13 (27.7%) | 0.16 |
| Male | 774 (62.2%) | 34 (72.3%) |  |
| Smoking, no. (%) | Missing n=29 | Missing n=1 |  |
| No smokers | 510 (41.9%) | 18 (39.1%) | 0.01 |
| Current smokers | 265 (21.8%) | 3 (6.5%) |  |
| Ex-smokers | 441 (36.3%) | 25 (54.3%) |  |
| **Comorbidities** |  |  |  |
| Arterial hypertension, no. (%) | Missing n=159 | Missing n=11 |  |
| No | 532 (49.0%) | 10 (27.8%) | 0.01 |
| Yes | 554 (51.0%) | 26 (72.2%) |  |
| Diabetes, no. (%) |  |  |  |
| No | 927 (74.5%) | 34 (72.3%) | 0.74 |
| Yes | 318 (25.5%) | 13 (27.7%) |  |
| Chronic heart disease, no. (%) |  |  |  |
| No | 844 (67.8%) | 24 (51.1%) | 0.02 |
| Yes | 401 (32.2%) | 23 (48.9%) |  |
| Chronic renal disease, no. (%) | Missing n=2 | Missing n=0 |  |
| No | 1090 (87.6%) | 35 (74.5%) | 0.01 |
| Yes | 155 (12.4%) | 12 (25.5%) |  |
| Chronic liver disease, no. (%) |  |  |  |
| No | 1,201 (96.5%) | 46 (97.9%) | 0.61 |
| Yes | 44 (3.5%) | 1 (2.1%) |  |
| Chronic respiratory disease, no. (%) | Missing n=2 | Missing n=0 |  |
| No | 855 (68.8%) | 32 (68.1%) | 0.92 |
| Yes | 388 (31.2%) | 15 (31.9%) |  |
| **Radiologic findings** |  |  |  |
| Bilateral infiltrates, no. (%) | Missing n=2 | Missing n=1 |  |
| No | 1014 (81.6%) | 28 (60.9%) | <0.01 |
| Yes | 229 (18.4%) | 18 (39.1%) |  |
| **Severity** |  |  |  |
| SpO2/FiO2 ratio at admission (IQR) | 441.4 (416.2-452.4) | 369.5 (293.6-423.8) | <0.01 |
| CURB-65 score (IQR) | 1 (1-2) | 2 (2-3) | <0.01 |
| **Biochemical parameters at admission** |  |  |  |
| Urea, mg/dL (IQR) | 23 (15.6-35) | 36.6 (24-51.5) | <0.01 |
| C-reactive protein, mg/L (IQR) | 167.6 (82.6-282.1) | 207.2 (89.0-352.8) | 0.23 |
| Leucocyte count, cells/mL (IQR) | 13230 (9540-17700) | 11520 (8100-18300) | 0.56 |
| Neutrophil count, cells/mL (IQR) | 10874.0 (7344.5-15115.9) | 9589.3 (5405.1-17000.7) | 0.60 |
| Lymphocyte count, cells/mL (IQR) | 991.3 (671.5-1506.0) | 690.0 (380.7-1364.0) | 0.01 |
| Platelet count, 10^3^ cells/mL (IQR) | 216 (170-280) | 218 (153-278) | 0.82 |

Data are summarized as n (%) or median (interquartile range). Abbreviations: COPD, chronic obstructive pulmonary disease; ILD, interstitial lung disease; IQR, interquartile range; SpO2/FiO2, peripheral blood oxygen saturation/fraction of inspired oxygen. Continuous variables are compared using the Wilcoxon rank-sum and categorical variables are compared using Pearson's chi-squared test.

Supplementary Table 5. Baseline characteristics of CAP cohort according to abnormal markers.

| **Variables** | **Normal markers n=57** | **One abnormal marker n=404** | **Two abnormal markers n=588** | **All abnormal markers n=243** | **P-value** |
| --- | --- | --- | --- | --- | --- |
| **Demographics** |  |  |  |  |  |
| Age (IQR) | 67 (54-78) | 68 (51-79) | 73 (61-81) | 74 (64-83) | <0.01 |
| Sex, no. (%) |  |  |  |  |  |
| Female | 20 (35.1%) | 162 (40.1%) | 219 (37.2%) | 83 (34.2%) | 0.48 |
| Male | 37 (64.9%) | 242 (59.9%) | 369 (62.8%) | 160 (65.8%) |  |
| Smoking, no. (%) |  |  |  |  |  |
| No smokers | 26 (47.3%) | 184 (46.8%) | 219 (38.1%) | 99 (41.4%) | <0.01 |
| Current smokers | 11 (20.0%) | 86 (21.9%) | 138 (24.0%) | 33 (13.8%) |  |
| Ex-smokers | 18 (32.7%) | 123 (31.3%) | 218 (37.9%) | 107 (44.8%) |  |
| **Comorbidities** |  |  |  |  |  |
| Arterial hypertension, no. (%) |  |  |  |  |  |
| No | 26 (51.0%) | 209 (55.3%) | 223 (44.4%) | 84 (44.0%) | 0.01 |
| Yes | 25 (49.0%) | 169 (44.7%) | 279 (55.6%) | 107 (56.0%) |  |
| Diabetes, no. (%) |  |  |  |  |  |
| No | 41 (71.9%) | 325 (80.4%) | 412 (70.1%) | 183 (75.3%) | <0.01 |
| Yes | 16 (28.1%) | 79 (19.6%) | 176 (29.9%) | 60 (24.7%) |  |
| Chronic heart disease, no. (%) |  |  |  |  |  |
| No | 38 (66.7%) | 282 (69.8%) | 402 (68.4%) | 146 (60.1%) | 0.07 |
| Yes | 19 (33.3%) | 122 (30.2%) | 186 (31.6%) | 97 (39.9%) |  |
| Chronic renal disease, no. (%) |  |  |  |  |  |
| No | 51 (89.5%) | 364 (90.1%) | 507 (86.2%) | 203 (83.5%) | 0.08 |
| Yes | 6 (10.5%) | 40 (9.9%) | 81 (13.8%) | 40 (16.5%) |  |
| Chronic liver disease, no. (%) |  |  |  |  |  |
| No | 54 (94.7%) | 393 (97.3%) | 566 (96.3%) | 234 (96.3%) | 0.71 |
| Yes | 3 (5.3%) | 11 (2.7%) | 22 (3.7%) | 9 (3.7%) |  |
| Chronic respiratory disease, no. (%) |  |  |  |  |  |
| No | 38 (66.7%) | 290 (72.0%) | 395 (67.3%) | 164 (67.5%) | 0.42 |
| Yes | 19 (33.3%) | 113 (28.0%) | 192 (32.7%) | 79 (32.5%) |  |
| **Radiologic findings** |  |  |  |  |  |
| Bilateral infiltrates, no. (%) |  |  |  |  |  |
| No | 52 (91.2%) | 343 (84.9%) | 472 (80.3%) | 175 (72.9%) | <0.01 |
| Yes | 5 (8.8%) | 61 (15.1%) | 116 (19.7%) | 65 (27.1%) |  |
| **Severity** |  |  |  |  |  |
| SpO2/FiO2 ratio at admission (IQR) | 457.14 (452.38-461.90) | 456.19 (447.62-461.90) | 431.43 (407.14-442.86) | 423.81 (378.57-439.05) | <0.01 |
| CURB-65 score (IQR) | 1 (0-2) | 1 (0-2) | 2 (1-2) | 2 (1-2) | <0.01 |
| **Biochemical parameters at admission** | |  |  |  |  |
| Urea, mg/dL (IQR) | 18.5 (14-25) | 18 (13.5-28) | 25 (17-37.3) | 29.9 (20.8-45.6) | <0.01 |
| C-reactive protein, mg/L (IQR) | 35.7 (20.7-48.2) | 124.4 (48.3-254.1) | 190.2 (102.8-288.8) | 244.0 (145.4-326.0) | <0.01 |
| Leucocyte count, cells/mL (IQR) | 10500 (7600-15900) | 13520 (9855-17685) | 14025 (10415-18830) | 10490 (7040-15550) | <0.01 |
| Neutrophil count, cells/mL (IQR) | 7714.7 (5350.5-13660.4) | 10879.6 (7327.0-14973.3) | 11732.2 (8276.3-16066.6) | 9048. (5970.4-13620.2) | <0.01 |
| Lymphocyte count, cells/mL (IQR) | 1234.2 (974.9-1608.9) | 1212.3 (916.9-1686.9) | 1061.3 (769.5-1541.8) | 530.0 (392.0-640.0) | <0.01 |
| Platelet count, 10^3^ cells/mL (IQR) | 213 (165-280) | 220.5 (173.5-290) | 226 (176-299) | 190 (151-241) | <0.01 |

Data are summarized as n (%) or median (interquartile range). Abnormal markers: lymphocyte count < 724 cells/mm^3^, CRP > 60 mg/L, SpO2/FiO2 < 450. Abbreviations: IQR, interquartile range; SpO2/FiO2, peripheral blood oxygen saturation/fraction of inspired oxygen. Continuous variables are compared using the Kruskal-Wallis test and categorical variables are compared using Pearson's chi-squared test.

Supplementary Table 6. COVID-19 and CAP data association tables for in-hospital mortality and penalised logistic regression analysis adjusted for age, sex and CURB65.

| **COVID-19 cohort** | | | | |
| --- | --- | --- | --- | --- |
| **Biomarkers** | **Events** | **N** | **% Deaths (CI 95%)** | ***OR (95% CI)** |
| Normal markers | 15 | 358 | 4.2% (2.5; 6.8) | - |
| One abnormal marker | 47 | 440 | 10.7% (8.1; 13.9) | 1.69 (0.90;3.20) |
| Two abnormal markers | 78 | 419 | 18.6% (15.2; 22.6) | 2.70 (1.47;4.98) |
| All abnormal markers | 75 | 235 | 31.9% (26.3; 38.2) | 5.17 (2.76;9.73) |
| Total | 215 | 1452 | 14.8% (13.1; 16.7) | - |
| **CAP cohort** | | | | |
| Normal markers | 0 | 57 | 0.0% (0.0; 6.2) | – |
| One abnormal marker | 9 | 404 | 2.2% (1.0; 4.2) | 2.61 (0.15;46.17) |
| Two abnormal markers | 17 | 588 | 2.9% (1.7; 4.6) | 2.38 (0.14;40.85) |
| All abnormal markers | 21 | 243 | 8.6% (5.4; 12.9) | 6.30 (0.37;107.77) |
| Total | 47 | 1292 | 3.6% (2.7; 4.8) | – |

Penalized logistic regression model performed and adjusted by sex and age. Abbreviations: CI, confidence interval; ICU, intensive care unit; OR, odds ratio. *Adjusted by sex, age and CURB65. AUROCs COVID-19 cohort 0.85 (0.82;0.87); CAP cohort 0.79 (0.72;0.86).

**Supplementary Figures**

**
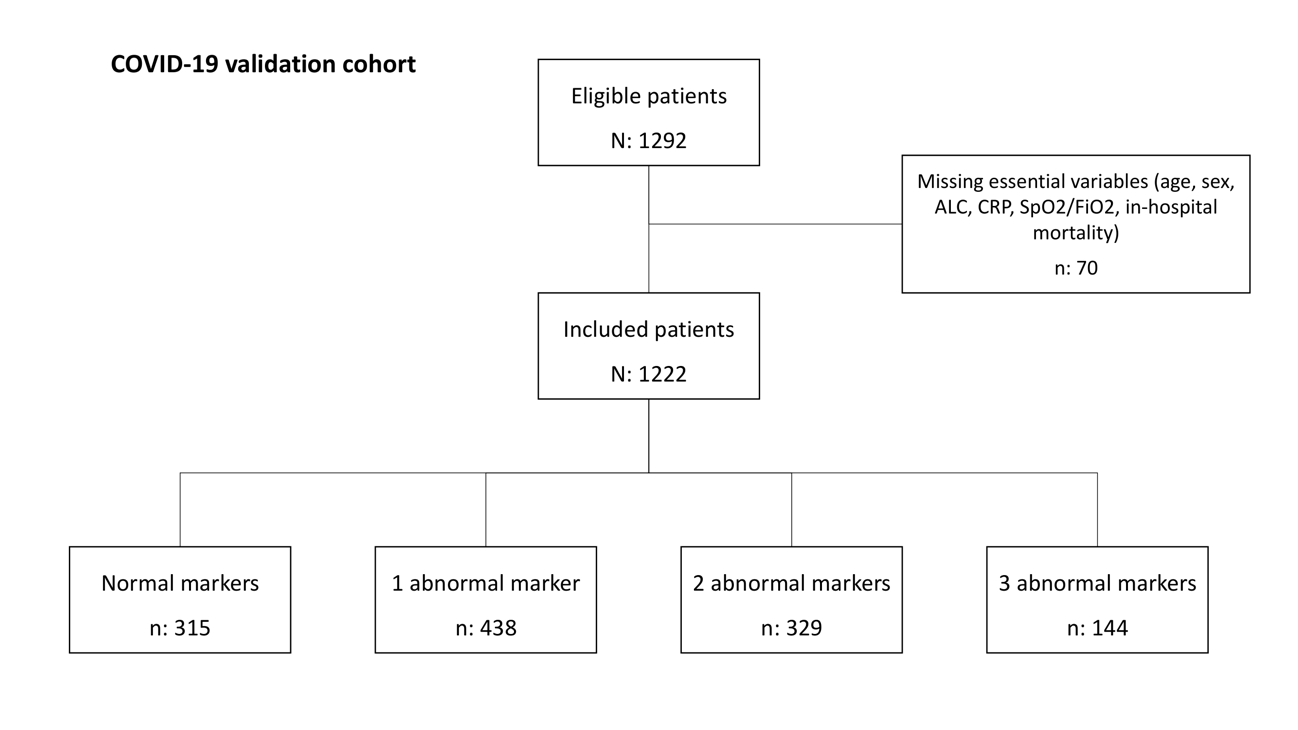
**

**Supplementary Figure 1. Flowchart of COVID-19 validation cohort.**

ALC, absolute lymphocyte count; CRP, C-reactive protein; SpO2/FiO2, peripheral blood oxygen saturation/fraction of inspired oxygen.


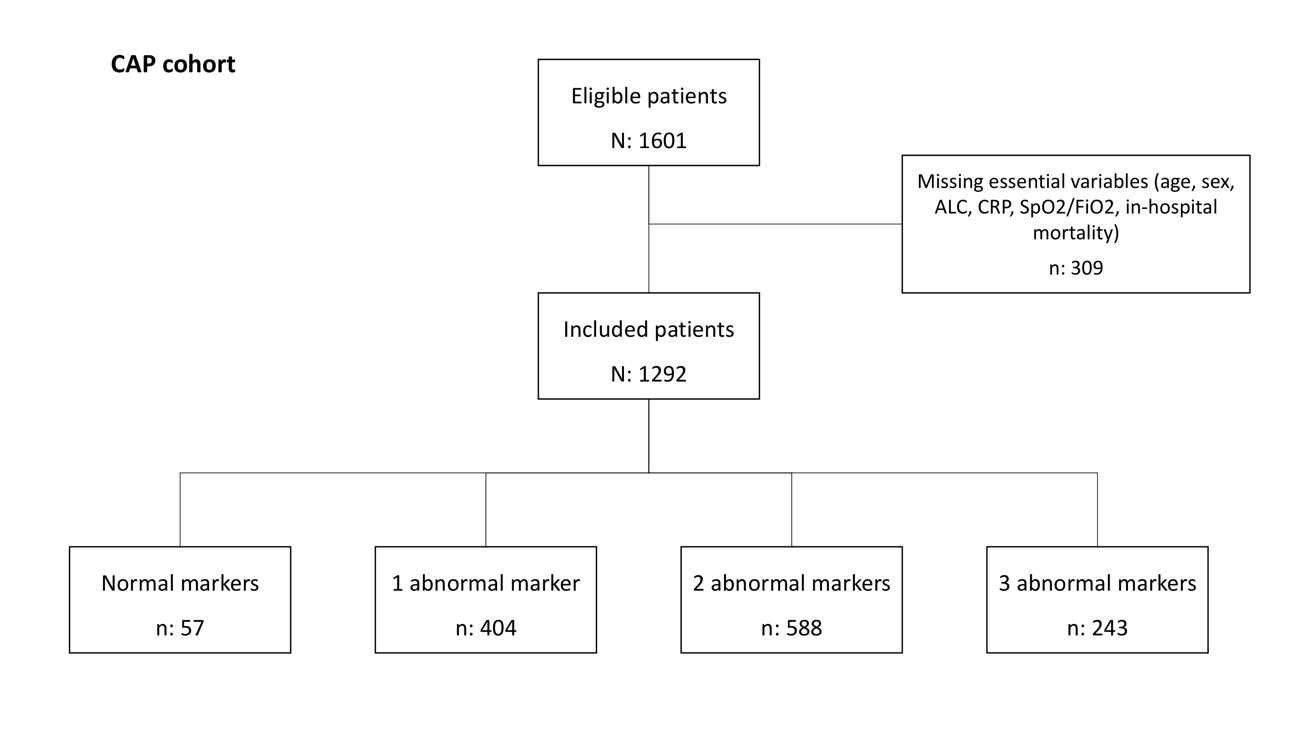


**Supplementary Figure 2. Flowchart of CAP cohort.**

ALC, absolute lymphocyte count; CAP, community-acquired pneumonia; CRP, C-reactive protein; SpO2/FiO2, peripheral blood oxygen saturation/fraction of inspired oxygen.
